# From Microbial Genomics to Metagenomics

**DOI:** 10.1155/2020/9357450

**Published:** 2020-05-31

**Authors:** Ravi Kant, Abhishek Kumar, Tarja Sironen

**Affiliations:** ^1^Department of Veterinary Biosciences, Faculty of Veterinary Medicine, University of Helsinki, Helsinki, Finland; ^2^Department of Virology, Faculty of Medicine, University of Helsinki, Helsinki, Finland; ^3^Institute of Bioinformatics, International Technology Park, Bangalore 560066, India; ^4^Manipal Academy of Higher Education (MAHE), Manipal, 576104 Karnataka, India

In the last 15 years, rapid application of next-generation sequencing (NGS) technologies has revolutionized the practice of microbial science. NGS has provided an unprecedented view on microbial diversity, and NGS technologies have allowed us to investigate complex microbial ecosystems, such as the human gastrointestinal (GI) microbiota, which consists of over three million genes from mainly Gram-positive bacteria [1–3].

Genomic comparisons of different bacterial genera and species have helped reveal the evolutionary origins of virulence and niche specification. Comparative analyses that compile the genomes of different strains from the same or different species (into what is called a “pangenome”) have revealed that the gene content within an entire species or genera is much more than that of a single strain or species. Moreover, this sort of study has aided the understanding of one of the dominant genetic forces behind bacterial evolution, specifically the concept of lateral gene transfer between microorganisms [4, 5].

Steady advances in sequencing technologies have allowed us to elucidate the genetics of microbial interactions, for example, through comparative metagenomic and metatranscriptomic analyses of bacterial communities. Metagenomics is one of the most rapidly growing fields in the microbial sciences. A metagenomic approach provides an extraordinary view of the diverse microbial world in different environments, such as in human and animal body sites, marine and other water bodies, soil, and air. Metagenomic and metatranscriptomic analyses of bacterial communities can reveal the genetics of microbial interactions and have been widely used by researchers in diverse disciplines such as ecology, energy, agriculture, biotechnology, and medicine [6]. As a rapidly growing field, comparative genomics and metagenomics also present many challenges that must to be addressed.

For our special issue, we received several manuscripts and, through rigorous review, selected five for publication. In “*Streptococcus halichoeri*: Comparative Genomics of an Emerging Pathogen,” K. Aaltonen et al. performed whole-genome sequencing of 20 different strains of an emerging pathogen, *S. halichoeri*, using the Illumina MiSeq platform and performed annotation using an automatic annotation pipeline RAST. The authors performed extensive all-against-all comparisons to unravel the core and pangenomes. Their findings highlight that *S. halichoeri* is a highly variable species with several virulence factors that indicate potential for significant pathogenicity. They also observed very little host species-specific markers in the genomes but instead observed a loose clustering according to species, as though adaptation is still incomplete. This suggests that the host switches into dogs, humans, and fur animals were rather recent and ongoing and possibly coincided with the beginning of the Fur Animal Epidemic Necrotic Pyoderma (FENP) epidemic. The authors also postulated that this species may have a marine origin as adhesins are the largest single category of virulence factors from the core genome.

In “Comparative Genomics of *Actinobacillus pleuropneumoniae* Serotype 8 Reveals the Importance of Prophages in the Genetic Variability of the Species,” I. G. de Oliveira Pardo et al. presented the genome of *A. pleuropneumoniae* serotype 8 along with comparisons of seven genomes of seven serotype 8 clinical isolates with the other genomes of 12 serotypes. *A. pleuropneumoniae* is the causative agent of porcine pleuropneumonia. Serotype 8 is the most widely distributed in the United States, Canada, United Kingdom, and southeastern Brazil. The proposed genomic analyses of serotype 8 genomes resulted in a set of 2352 protein-coding sequences; 76.6% of these proteins are commonly shared across all serotypes, 18.5% are shared with some serotypes, and 4.9% were differentially present, which are primarily a series of hypothetical and regulatory mobile elements. Additionally, the authors identified 30 prophage sequences, of which 16 are members of the family Pasteurellaceae. This suggests that mobile genetic elements play a role in the diversity and evolution of *A. pleuropneumoniae*.

In “Genomic Analysis of *Bacillus megaterium* NCT-2 Reveals Its Genetic Basis for the Bioremediation of Secondary Salinization Soil,” B. Wang et al. reported genome sequencing of a nitrate-uptake bacterium *B. megaterium* NCT-2 using HiSeq and PacBio sequencing. The total size of this genome is 5.88 Mbp with 37.87% GC content including 10 indigenous plasmids. The *B. megaterium* NCT-2 genome contains 5606 genes, 142 tRNAs, and 53 rRNAs. The authors also described genes involved in bioremediation in secondary salinization soil.

*Streptococcus parauberis* is a Gram-positive, alpha-haemolytic lactic acid coccoid-shaped bacterium. This bacterium is a fish pathogen that especially affects olive flounder (*Paralichthys olivaceus*). Accordingly, *S. parauberis* is responsible for massive losses for fish farmers across different countries in Asia and Europe. *S. parauberis* is known to possess antibiotic resistance against most antibacterial drugs (such as tetracycline, oxytetracycline, and erythromycin) that would be used to treat this pathogen. Identification of alternative therapeutic agents against *S. parauberis* is therefore necessary. In “Pharmacodynamics of Ceftiofur Selected by Genomic and Proteomic Approaches of *Streptococcus parauberis* Isolated from the Flounder, *Paralichthys olivaceus*,” N. Boby et al. employed an integrative multiomic strategy to present subtractive and comparative metabolic and genomic-based findings of therapeutic targets against *S. parauberis*. The authors also proposed ceftiofur as a new antimicrobial drug for treating *P. olivaceus* infected with *S. parauberis* by coupling multiomic approaches with pharmacodynamic profiles of the approved antimicrobial drugs.

In “FcircSEC: An R Package for Full Length circRNA Sequence Extraction and Classification,” T. Hossain et al. presented an R package for deciphering and classifying the circRNA as FcircSEC (Full-Length circRNA Sequence Extraction and Classification). All existing tools for circRNA predictions only provide genomic coordinates of the predicted circRNA. Hence, the authors developed an R package that focused on several features, including gene annotation. This tool is capable of genomic location-based classification of circRNA, such as exonic or intronic.

This R-based tool is capable of handling datasets of several species including human data. The authors validated the resulting data using three different databases, namely, circBase, circRNADb, and PlantcircBase. This R tool FcircSEC is based on the Bioconductor package Biostring and uses the output from state-of-the-art circRNA prediction tools. The R package FcircSEC is freely available at its dedicated website (http://hpcc.siat.ac.cn/FcircSEC/Home.html), where the authors provided downloadable datasets, a reference manual, source code, and Windows binaries. The authors followed the standards of software sharing by providing this R tool at the Comprehensive R Archive Network (CRAN, https://cran.r-project.org/web/packages/FcircSEC/index.html) and also at the GitHub repository (https://github.com/tofazzal4720/FcircSEC).

Four out of five articles in this special issue are focused on comparative genomics, and one is focused on the development of a novel method. In our opinion, the articles in this special issue would allow us to explore new approaches to understand the molecular mechanisms linked to microbial function. We hope that these materials will assist and inspire readers working in the field of microbial genomics.
